# Brain–computer interface use is a skill that user and system acquire together

**DOI:** 10.1371/journal.pbio.2006719

**Published:** 2018-07-02

**Authors:** Dennis J. McFarland, Jonathan R. Wolpaw

**Affiliations:** National Center for Adaptive Neurotechnologies, Wadsworth Center, Albany, New York, United States of America

## Abstract

A brain–computer interface (BCI) is a computer-based system that acquires, analyzes, and translates brain signals into output commands in real time. Perdikis and colleagues demonstrate superior performance in a Cybathlon BCI race using a system based on “three pillars”: machine learning, user training, and application. These results highlight the fact that BCI use is a learned skill and not simply a matter of “mind reading.”

A brain–computer interface (BCI) is a computer-based system that acquires, analyzes, and translates brain signals into output commands in real time [1]. BCIs are distinctive in that they do not use the brain’s normal output pathways of peripheral nerves and muscles. BCI studies have focused mainly on restoring communication and control to people paralyzed by chronic neuromuscular disorders, such as amyotrophic lateral sclerosis (ALS), brainstem stroke, or high-level spinal cord injury. Most of these studies have been demonstrations of proof of principle with able-bodied users. Long-term independent use of BCIs for communication or control by individuals who can actually benefit from them is limited to a handful of case reports [2–6] and one group study [7].

One prominent class of BCIs use sensorimotor rhythms (SMRs). SMRs are oscillations (i.e., mu [8–12 Hz] and beta [18–30 Hz]) in electroencephalographic (EEG) activity recorded over sensorimotor cortices; they change in amplitude with movement, imagined movement, or preparation for movement. People can also learn through a training protocol to increase or decrease SMR amplitude and can use this control to move a computer cursor or operate another device [8–10]. Furthermore, they can learn to use several different SMRs to control movement in multiple dimensions simultaneously and to support sequential mouse-like control [11–15].

The performance of BCIs that use SMRs (as well as that of BCIs that use cortical neurons [16]) improves with training. Their usage is in large part a skill that is acquired through practice [1]. The BCI user learns to encode his/her intent in brain signal features (e.g., SMRs) that the BCI can record, extract, and translate into output commands. Thus, BCI operation entails the effective cooperation of two adaptive controllers: the user, who seeks to encode his/her intent in brain signals that the BCI can recognize; and the BCI, which adapts to recognize these signals and translate them into the intended output commands.

Many BCI studies have largely ignored the user-training component of BCI operation. They have focused almost exclusively on machine learning (e.g., on the BCI learning how to recognize the brain signals associated with particular types of motor imagery). This popular trend probably results from an emphasis on the inadequate notion that BCI users are simply executing pre-existing cognitive tasks such as motor imagery [17–18] (see Box 1).

**Box 1. BCI use as an acquired skill**

It is often assumed that the way to train users with a sensorimotor rhythm (SMR)-based BCI is to ask them to generate specific mental states through motor imagery [18]. Different BCI commands are often linked to different imagery (e.g., imagine hand movement to move the cursor up and foot movement to move it down). However, as the user’s SMR control improves, and particularly when users advance to controlling multiple dimensions, users tend to abandon the use of imagery [14]. While motor imagery may provide a logical and effective starting point for user training, it becomes unnecessary and may even be an impediment as training progresses. For example, Kober and colleagues [23] found that those subjects who reported using no specific mental strategy after 10 SMR training sessions showed improved performance. In contrast, subjects reporting various mental strategies after 10 training sessions showed no improvement. These results suggest that successful SMR control after extended training involves implicit learning mechanisms. Thus, SMR-based BCI control after extended training resembles typical motor performance in that it tends to become automatic (i.e., implicit) with practice. Indeed, studies in animals show that BCI control involves plasticity in corticostriatal systems typically involved in motor control [24]. Viewing SMR control as similar to other forms of motor control suggests using principles of motor learning in designing SMR-based BCI applications and in training people to use them. Motor learning involves both explicit and implicit processes, which have different characteristics [25]. Explicit instructions can interfere with implicit processes, particularly in well-trained people [26].

In contrast, the article by Perdikis and colleagues [19] in this issue illustrates the importance of recognizing and facilitating both adaptive aspects of BCI operation: adaptation by the user and adaptation by the BCI. They describe their SMR-based BCI training of two people with tetraplegia to compete in the Cybathlon BCI race; these two users went on to dominate the competition. The authors ascribe this success to a mutual learning approach that gives equal importance to “three pillars”: machine learning, user training, and application. They note that the other entries in the Cybathlon relied predominately on machine learning. They assert that their success supports the view that “BCI is a skill to be learned.”

As noted above, the fact that BCI use is a skill has been emphasized previously, and Perdikis and colleagues [19] acknowledge that their conclusions are limited by the fact that they are based on an uncontrolled study of only two individuals. Nevertheless, their study is distinctive and notable because the BCI users were severely disabled and, most important, because it reports their performance in a highly competitive and stressful setting (rather than simply in a protected laboratory environment). Given these considerations, the results provide impressive new evidence of the effectiveness of a mutual learning approach.

One of the users in the Perdikis and colleagues [19] study had the best overall performance but did not do well (i.e., “choked”) in the final competition. This finding may illustrate an important feature of skilled performance: the focus of attention can be critical. Studies of motor performance show that an external focus of attention to the outcome of an action results in performance that is superior to that associated with an internal focus on the mechanics of the action [20]. Indeed, Schucker and colleagues [21] report that individuals who choked under pressure reported greater attention to the details of their movements (i.e., allocation of attention to the different body parts that were involved in movement execution). It may be the case that this BCI user was a victim of an internal focus of attention. Ultimately, the effectiveness of various strategies for BCI control should clarify the nature of this type of skill and lead to methods for improving user performance.

Perdikis and colleagues [19] also discuss the difficult problem of mutual learning: the ongoing adaptive interaction of the user’s brain, which produces the signals measured by the BCI, and the BCI itself, which translates these signals into commands [1,22]. Although there has been considerable work on the design of controllers that involve a single adaptive element, coadaptation in BCI design represents an important new challenge.

Future BCI studies would do well to consider the three pillars of mutual learning stressed by Perdikis and colleagues [19]. More emphasis on user learning and application design are likely to move this exciting area of research forward. The ultimate goal should be the development of fast and accurate BCI systems that can be reliably operated by the individuals with severe neuromuscular disabilities who can benefit from their use.

## Abbreviations

ALS: amyotrophic lateral sclerosis
BCI: brain–computer interface
EEG: electroencephalographic
SMR: sensorimotor rhythm
